# Optimal Design of Annular Phased Array Transducers for Material Nonlinearity Determination in Pulse–Echo Ultrasonic Testing

**DOI:** 10.3390/ma13235565

**Published:** 2020-12-06

**Authors:** Sungjong Cho, Hyunjo Jeong, Ik Keun Park

**Affiliations:** 1 NDT Research Center, Seoul National University of Science and Technology, Seoul 01811, Korea; cho-sungjong@seoultech.ac.kr; 2 Department of Mechanical Engineering, Wonkwang University, Iksan 54538, Korea; 3 Department of Mechanical and Automotive Engineering, Seoul National University of Science and Technology, Seoul 01811, Korea; ikpark@seoultech.ac.kr

**Keywords:** phased array transducer, transducer optimization, beam focusing, pulse–echo mode, harmonic generation, stress-free boundary, total correction

## Abstract

Nonlinear ultrasound has been proven to be a useful nondestructive testing tool for micro-damage inspection of materials and structures operating in harsh environment. When measuring the nonlinear second harmonic wave in a solid specimen in the pulse–echo (PE) testing mode, the stress-free boundary characteristics brings the received second harmonic component close to zero. Therefore, the PE method has never been employed to measure the so-called “nonlinear parameter (*β*)”, which is used to quantify the degree of micro-damage. When there are stress-free boundaries, a focused beam is known to improve the PE reception of the second harmonic wave, so phased-array (PA) transducers can be used to generate the focused beam. For the practical application of PE nonlinear ultrasonic testing, however, it is necessary to develop a new type of PA transducer that is completely different from conventional ones. In this paper, we propose a new annular PA transducer capable of measuring *β* with improved second harmonic reception in the PE mode. Basically, the annular PA transducer (APAT) consists of four external ring transmitters and an internal disk receiver at the center. The focused beam properties of the transducers are analyzed using a nonlinear sound beam model which incorporates the effects of beam diffraction, material attenuation, and boundary reflection. The optimal design of the APAT is performed in terms of the maximum second harmonic reception and the total correction close to one, and the results are presented in detail.

## 1. Introduction

Power generation facilities in nuclear power and thermal power plants that are operated at high temperature and high pressure can lead to various types of micro-damage (e.g., deterioration, residual stress, fatigue, creep, and micro-cracks) as the number of years of use increases. The management of such damage is an essential part of ensuring the soundness and safe operation of power plants. In particular, a more reliable diagnosis technology is required for major components and parts made of nickel alloy or carbon steel welding because they are susceptible to micro-damage. 

Currently, in nondestructive testing of power generation facilities, conventional radiography testing (RT) is being replaced by ultrasonic testing. Among the various ultrasonic methods, phased array ultrasonic testing is widely applied to the inspection of power generation facilities and pressure vessels [1,2,3,4]. However, it is not easy to detect various types of micro-damage described above with conventional linear ultrasonic testing techniques. Nonlinear ultrasonic technology uses nonlinear acoustic effects that occur when a strong ultrasonic wave is incident inside a material. The nonlinear ultrasound, such as the second harmonic wave, is known to be sensitive to micro-damage [5,6,7,8,9,10], and mainly measures the nonlinear parameter, β, which is defined by the displacement amplitudes of the fundamental and second harmonic waves to quantify the degree of damage. Studies on nonlinear ultrasonic applications are actively being conducted [11,12,13,14,15,16,17,18,19,20], and the use of longitudinal waves dominates in most cases, although surface and Lamb waves are also used. Damage types include fatigue, deterioration, creep, and irradiation, and most studies measure the uncorrected nonlinear parameter, $\beta^{\prime }$, for ease and convenience of measurement.

Although nonlinear parameters are mainly measured in the through-transmission (TT) mode, pulse–echo (PE) measurements are frequently required for field applications. According to Bender et al. [21], the amplitude of the second harmonic received after reflection from the stress-free boundary of a sample in the PE mode is theoretically zero. This was the main reason the PE method has not been applied until recently. However, the zero reception of the second harmonic in the PE mode was the result of the pure plane wave. In the case of a real transducer of finite size, the second harmonic wave can be received owing to the diffraction effect, but it is extremely small and can only be measured when the specimen is thick enough [22,23]. Therefore, increasing the amplitude of the received second harmonic in applications of nonlinear PE method for thin samples is of utmost importance for obtaining the second harmonic signal with high signal-to-noise ratio and for accurate and reliable measurement of β.

It has been found that a focusing beam increases the amplitude of the received second harmonic in the PE testing of a sample with the stress-free boundary. Actually, the received second harmonic amplitude was found to significantly increase when a spherically focusing transducer was used in the water–air boundary [24,25]. The spherical focusing with a linear phased array ultrasonic transducer (PAUT) is not possible on the flat surface of a solid specimen. In addition, because the current PAUT for linear ultrasonic testing achieves beam focusing using dozens of channels and short pulses, it is still difficult to apply the nonlinear ultrasonic technique that employs a high-power toneburst type signal. It is also necessary to minimize the source nonlinearity, which is an important variable in nonlinear ultrasonic measurement. This is because when source nonlinearity occurs, it is mixed with the nonlinearity caused by damage, making it difficult to observe the damage-induced nonlinearity alone. Furthermore, the receive transducer should have a broad bandwidth capable of covering both fundamental and second harmonic wave frequencies. Therefore, for the development of PAUTs applicable to PE nonlinear testing, it is necessary to minimize the number of channels by designing a new type of PAUT that is completely different from the conventional PAUT. Before fabricating and applying such PAUT, a prototype design is required, and the focused beam properties should be fully understood. Further optimization of the PAUT is possible to achieve the maximum second harmonic reception and the uncorrected nonlinear parameter ($\beta^{\prime }$) close to the absolute nonlinear parameter (β).

In this paper, we propose an annular phased array transducer (APAT) and model the nonlinear acoustic fields generated by the APAT in the PE setup to determine the optimal dimensions of the transducer. Basically, the APAT for PE nonlinear testing purposes consists of the four external ring transmitters and an internal disk receiver at the center. The fundamental and second harmonic wave fields, which are focused at various positions of the 1 cm thick specimen and then reflected from the stress-free boundary, are calculated and their characteristics are examined with the received average fields. For a given specimen thickness and frequency, the optimization of the APAT is then performed in terms of the maximum possible reception of the second harmonic and the total correction as close to one as possible. The optimization results are presented in detail. The shape of the time domain waveform formed at the focal position and at the receiver position are examined through finite element (FE) simulation.

Section 2 describes the requirements of the phased array transducer to be considered when performing the nonlinear parameter measurement in the pulse–echo mode using a focused beam and introduces the conceptual design of an APAT along with its focal characteristics in linear ultrasound. In Section 3, we outline the nonlinear acoustic model developed in our previous work [26] and define the nonlinear parameter with necessary corrections. This model combines the effects of nonlinearity, diffraction, and boundary reflection in order to calculate the fundamental and second harmonic fields in the focused beam generated by the four annular transmit elements. Section 4 compares the focused beam properties of the two types of APAT—equal width (EW) and equal area (EA). We then present details on the optimization process of the EW type APAT and provide results on the received second harmonic amplitude and the relative nonlinear parameter. The FE simulation results are also presented in Section 4 to see how well the focused and/or received waveform matches the initially incident waveform. Conclusions are drawn in Section 5.

## 2. Phased Array Transducer Design 

**Figure 1:**
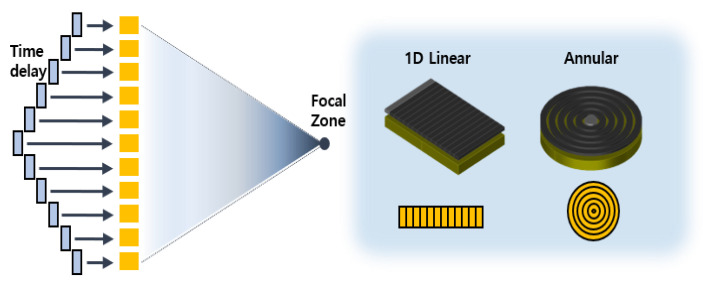
Schematic illustration of phased array beam focusing through time delay and two types of phased arrays.

Ultrasonic phased array techniques are widely used in nondestructive testing areas and in many medical applications. Some of the attractive features of PAs include electronic focusing and steering capabilities. To generate a focused beam at any specified angle and distance, time delays are calculated and applied electronically to each element, as shown in Figure 1. In general, PA types are classified as linear or annular depending on the shape and arrangement of the elements, as shown in Figure 1. 

To utilize the PA focusing technology to the measurement of nonlinear material properties, feasibility studies (e.g., senor materials and fabrication methods) and equipment availability must be preceded at the design stage of PAUT. Material nonlinearity measurements using the finite amplitude method require high power amplifiers, and the number of such amplifiers increases as the number of PA elements increases. Therefore, it is desirable to achieve beam focusing with minimum number of elements. 

The generation of second harmonic waves in solids typically requires very high input voltages at the transmitter. Therefore, it is important to minimize the source nonlinearity caused by the transmit element. Most transmit transducers use single crystal LiNbO_3_ instead of commercial transducers made of piezoelectric materials such as PZT for the purpose of harmonic generation with minimal source nonlinearity from the transmitter. The LiNbO_3_ piezoelectric element shows a narrowband spectrum around its fundamental resonant frequency when no backing material is used. Thus, a transducer made of LiNbO_3_ cannot receive both fundamental and second harmonic components at the same time. To solve this problem, the practical approach is to use a separate receive transducer of a broad bandwidth.

The next thing to consider is the calibration of the receiver. For a quantitative evaluation of the material damage, an absolute nonlinear parameter (*β*), not the uncorrected nonlinear parameter ($\beta^{\prime }$), needs to be measured. To measure *β*, the receive transducer must be calibrated [27,28,29]. To summarize the above, the PAUT for pulse–echo nonlinearity measurement requires a minimum number of transmit elements to generate a focused beam, separation of transmit and receive elements, and calibration of the receive element. 

**Figure 2:**
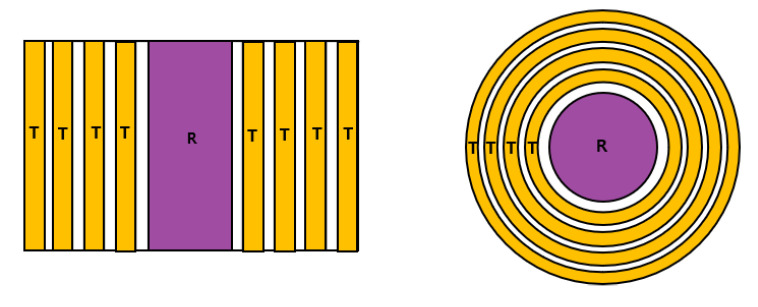
Configuration of linear and annular phase arrays.

Taking these requirements into account, the arrangement of the transmit and receive elements for the conceptual design of linear and annular PAs is shown in Figure 2. Compared with conventional PAs, the central element is used only for reception, and the other elements are used for transmission, where the transmit and receive elements have the same central axis. This design allows the application of conventional receiver calibration method. In the conceptual design and wave field simulation with a beam focusing, the number of transmit elements is limited to four. Because the central element is used for reception, the beam focusing behavior is analyzed along the central axis. The beam focusing simulation in this section was conducted using the CIVA program [30,31,32], a nondestructive simulation platform. In the CIVA simulation, aluminum was selected as the propagation medium, and the following acoustic properties including the nonlinear parameter were used [28]: longitudinal wave velocity, c=6422 m/s; density, $\rho =2700\;\mathrm{kg}/m^{3}$; fundamental wave frequency, f=5 MHz; and nonlinear parameter, β=5.5. The specifications of the PAUTs used in the simulation are summarized in Table 1. The specifications of the annular phased array were imported from the optimal design of the equal width (EW) type annular phased array described in Section 4. The diameter of the receive element is 3.2 mm, and the diameter of the innermost transmit element is 10 mm. Specifications of the EW phase array can be found in Table 2. The dimensions of the linear PA along the width direction are the same as the cross-sectional dimensions of the annular PA, and the length of the linear PA was taken arbitrarily as 10 mm. In Section 4, the effect of the sizes of the transmit elements (i.e., equal width type and equal area type) on the received amplitude of the fundamental wave and the second harmonic was compared.

**Figure 3:**
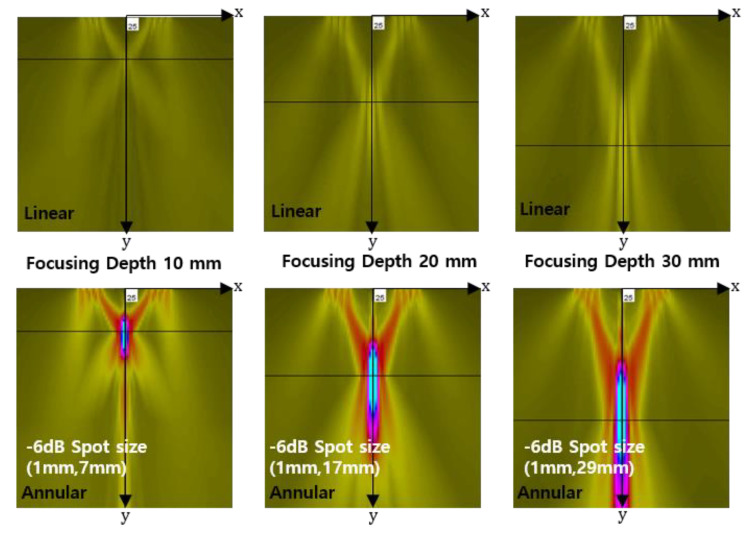
Beam focusing simulation results of the linear and annular phased arrays.

Beam focusing simulation results of the linear and annular phased arrays are shown in Figure 3. The simulations were performed in the linear ultrasound range. In the case of the linear PA, the focusing is hardly found, whereas, in the case of the annular PA, a distinct focusing can be seen at three focal lengths. This is due to the geometry of the annular PA, which is much more efficient in forming the focused beam. If we look at the results of the annular PA in more detail, the simulated focal spot sizes at −6 dB along the cross-axis of the beam are about 1 mm for all three focal lengths. If we treat the annular PA as a single transmit element of diameter *D*, the measured spot size is slightly larger than the estimated focal spot size using the equation d=λL/D where *λ* is the wavelength, *L* is the focal length, and *D* is the total width of the array. On the other hand, the focal spot size along the on-axis of the beam increases with increasing focal length, and this is clearly seen in the simulation results of Figure 3. Increased focal spot size also means decrease of peak amplitude. The difficulty in creating well-shaped focal zones in the annular PA can be attributed to the hollow structure and a small number of elements [33,34,35]. The current annular PA structure with four transmit elements is hollow in the center, but the overall beam focusing behavior is similar to that observed in the conventional linear PAs with dozens of elements. In particular, since it has a good focusing performance at the focal length of 10 mm, it can be used for pulse–echo nonlinear measurement of relatively thin specimens with a thickness of about 10 mm.

## 3. Annular PA Wave Fields and Definition of β

### 3.1. Theory

**Figure 4:**
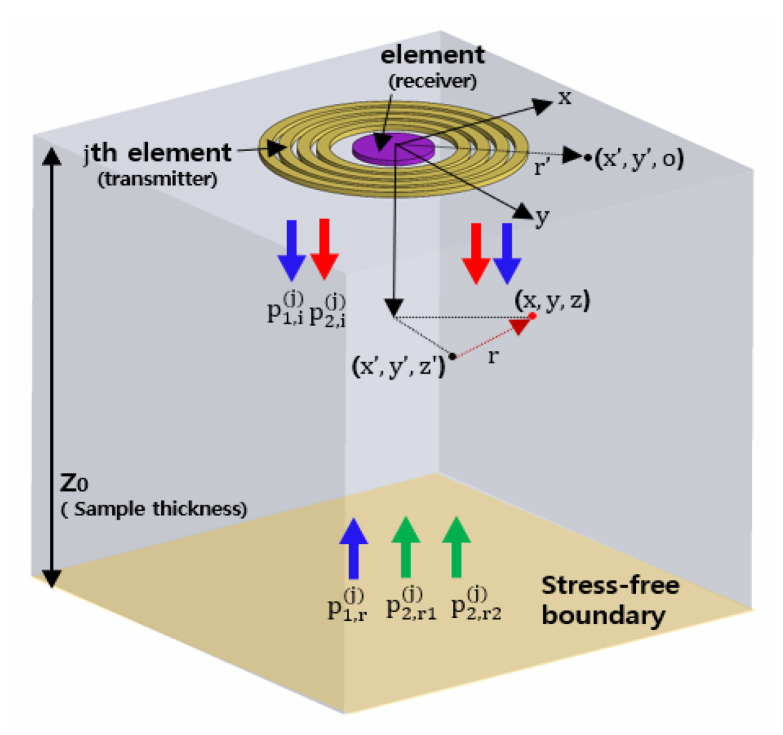
Schematic illustration of second harmonic generation process in a nonlinear PE testing with the stress-free boundary.

Second harmonic generation in the nonlinear PE testing with the stress-free boundary condition is schematically illustrated in Figure 4. The APAT consisting of four ring transmitters and a central disk receiver are also included. In Figure 4, $p_{1,i}$ is the acoustic pressure of the fundamental wave emitted from the ring transmitters, and $p_{2,i}$ is the generated second harmonic wave owing to the forcing of $p_{1,i}$. $p_{1,r}$ denotes the reflected fundamental wave when the wave $p_{1,i}$ hits the boundary, $p_{2,r1}$ is the reflected wave when the wave $p_{2,i}$ hits the boundary, and $p_{2,r2}$ is the second harmonic wave generated by the reflected $p_{1,r}$. Therefore, the total reflected second harmonic, $p_{2,r}$, can be obtained by adding $p_{2,r1}$ and $p_{2,r2}$. Both the reflected fundamental and second-harmonic waves are received by the center receiver.

### 3.2. Sound Beam Solution

The second harmonic wave is produced due to material nonlinearity when the finite amplitude fundamental wave radiates from the transmitter and propagates in the solid. The related acoustic fields for a single element circular transducer have been derived previously [26,36,37]. In this section, we briefly present the mathematical equations to calculate the received acoustic fields when a phased array transducer composed of four ring elements radiates a finite amplitude longitudinal wave. The incident fundamental wave $p_{1,i}$ and the generated second harmonic $p_{2,i}$ are given by Equations (1) and (2). Pressure *p* is used here as a field variable.

(1) $$p_{1,i}\left(x_{1},y_{1},z_{1}\right)=-2ik\overset{+\infty }{\underset{-\infty }{\int }}\overset{+\infty }{\underset{-\infty }{\int }}p_{1}\left(x^{\prime },y^{\prime },0\right)\;G_{1}(x,y,z|x^{\prime },y^{\prime },0)\;dx^{\prime }dy^{\prime }$$

(2) $$p_{2,i}\left(x_{1},y_{1},z_{1}\right)=\frac{2\beta k^{2}}{\rho c^{2}}\overset{z}{\underset{0}{\int }}\overset{+\infty }{\underset{-\infty }{\int }}\overset{+\infty }{\underset{-\infty }{\int }}p_{1}^{2}\left(x^{\prime },y^{\prime },z^{\prime }\right)\;G_{2}(x,y,z|x^{\prime },y^{\prime },z^{\prime })\;dx^{\prime }dy^{\prime }dz^{\prime }$$

where the Green’s function is given by the following equation:

(3) $$G_{1}(x,y,z|x^{\prime },y^{\prime },0)=\frac{1}{4\pi r}exp(ikr)$$

(4) $$G_{2}(x,y,z|x^{\prime },y^{\prime },z^{\prime })=\frac{1}{4\pi R}exp(i2kR)$$

Here, $\;r=\sqrt{{\left(x-x^{\prime }\right)}^{2}+{\left(y-y^{\prime }\right)}^{2}+z^{2}}$ and $R=\sqrt{{\left(x-x^{\prime }\right)}^{2}+{\left(y-y^{\prime }\right)}^{2}+{\left(z-z^{\prime }\right)}^{2}}$. For $p_{1}$, the source function is $p_{1}\left(x^{\prime },y^{\prime },z^{\prime }=0\right)$, and the integration is applied over the transducer surface element $ds^{\prime }=dx^{\prime }dy^{\prime }$ at the source plane $z^{\prime }=0$,

(5) $$p_{1}\left(x^{\prime },y^{\prime },z^{\prime }=0\right)=p_{0},\;\;a^{2}\leq x^{\prime 2}+y^{\prime 2}\leq b^{2}$$

where $p_{0}$ is the uniform acoustic pressure and a and b are the inner and outer radii of the ring transmitter. $p_{1,i}$ and $p_{1,r}$ of the *m*th transmission element can be obtained by calculating $p_{1,i}^{\left(m\right)}$ and $p_{1,r}^{\left(m\right)}$ under different boundary conditions of Equation (5), and the total fields are found by adding them together. The reflected fundamental pressure $p_{1,r}$ is given by

(6) $$p_{1,r}\left(x,y,z\right)=R_{1}p_{1i}\left(x,y,z\right)$$

The total pressure of the reflected second harmonic $p_{2,r}$ is obtained as the sum of $p_{2,r1}$ and $p_{2,r2}$ given by

(7) $$p_{2,r1}\left(x,y,z\right)=-2ik\overset{\infty }{\underset{-\infty }{\int }}\overset{\infty }{\underset{-\infty }{\int }}R_{2}p_{2,i}\left(x^{\prime },y^{\prime },z_{0}\right)G_{2}(x,y,z|x^{\prime }y^{\prime },z_{0})\;dx^{\prime }dy^{\prime }$$

(8) $$p_{2,r2}\left(x,y,z\right)=\frac{2\beta k^{2}}{\rho c^{2}}\overset{z}{\underset{z_{0}}{\int }}\overset{\infty }{\underset{-\infty }{\int }}\overset{\infty }{\underset{-\infty }{\int }}{\left\{p_{1,r}\left(x^{\prime },y^{\prime },z^{\prime }\right)\right\}}^{2}G_{2}(x,y,z|x^{\prime },y^{\prime },z^{\prime })\;dx^{\prime }dy^{\prime }dz^{\prime }$$

In Equations (6) and (7), *R*_1_ and *R*_2_ are the reflection coefficients for the fundamental and second harmonic waves at the solid–air interface and are given by $R_{1}=R_{2}=-1$. The reflected second harmonic fields for *m*th element can be obtained by calculating $p_{2,r1}^{\left(m\right)}$ and $p_{2,r2}^{\left(m\right)}$, and the total fields are found by adding contributions from all elements. 

Next, to calculate the received pressure at a distance z by the receiver of area $S_{R}$, the concept of the average pressure can be defined and calculated as follows:

(9) $${\tilde{p}}_{n}\left(z\right)=\frac{1}{S_{R}}\underset{S_{R}}{\int }p_{n}\left(x,y,z\right)dS_{R}\;\;\;\;\;\;\;\;\;\;\;\;\;n=1,2$$

### 3.3. Time Delay

**Figure 5:**
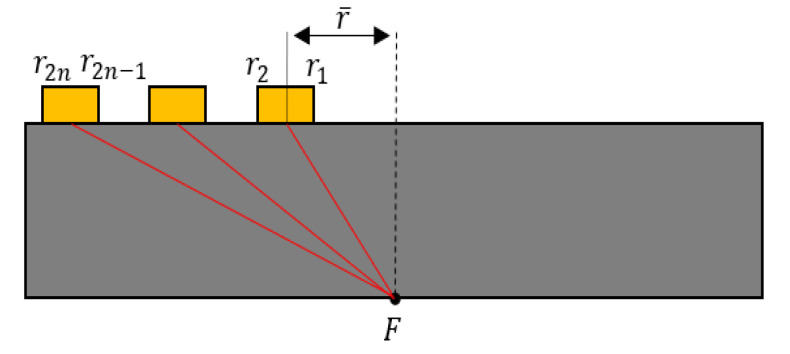
Geometrical parameters for calculating the focusing time delay of phased array.

Consider an array of *N* elements radiating into a solid to produce a sound beam with a focal length *F*, as shown in Figure 5. The focusing time delays can be calculated as follows [38]:

(10) $$\Delta t_{N}=\frac{\sqrt{{\left(\frac{r_{2N}+r_{2N-1}}{2}\right)}^{2}+F^{2}}-F}{c}=\frac{\sqrt{{\bar{r_{N}}}^{2}+F^{2}}-F}{c}$$

where $\Delta t_{N}$ is the required time delay for element *N* = 0, 1, …, *N*. Note that in Equation (10) each calculated time has a positive value, which is a time delay. The delay of the time-domain signal is equivalent to multiplying the frequency domain signal by a phase term that is linear in frequency and proportional to its delay. If F(ω) is the Fourier transform of the time domain signal f(t), then the Fourier transform of the time-shifted signal $f\left(t-\Delta t_{N}\right)$ can be obtained as $\exp\left(i\omega \Delta t_{N}\right)F\left(\omega \right)$, where $\Delta t_{N}$ is the delay time.

### 3.4. Definition of β with Total Correction

Equation (9) can be expressed more conveniently in terms of the plane wave solutions modified by the correction terms owing to the effects of attenuation, diffraction, and boundary reflection, i.e.,

(11) $${\tilde{p}}_{1,r}=\left[p_{1}^{plane}\left(z\right)\right]\left[C_{T1}\right]$$

(12) $${\tilde{p}}_{2,r}=\left[p_{2}^{plane}\left(z\right)\right]\left[C_{T2}\right]$$

Here, $p_{1}^{plane}=p_{0}exp(ikz)$ and $p_{2}^{plane}=\frac{\beta kp_{0}^{2}z}{2\rho c^{2}}exp(2ikz)$ where *k* is the wave number, ρ is the density, and *c* is the wave velocity. In addition, the average pressure is calculated at the initial source position, i.e., at the total propagation distance $z=2z_{0}$. In Equations (11) and (12), $C_{Tn}$ is the correction due to attenuation, diffraction, and boundary reflection in the fundamental (*n* = 1) and second harmonic (*n* = 2) waves and is defined as follows:

(13) $$C_{T1}=R_{1}M_{1}{\tilde{D}}_{1}$$

(14) $$C_{T2}=\left[R_{2}M_{21}{\tilde{D}}_{21}+R_{1}^{2}M_{22}{\tilde{D}}_{22}\right]$$

where $M_{1}$, $M_{21}$, and $M_{22}$ and ${\tilde{D}}_{1}$, ${\tilde{D}}_{21}$, and ${\tilde{D}}_{22}$ are the attenuation corrections and diffraction corrections in ${\tilde{p}}_{1,r}$, ${\tilde{p}}_{2,r1}$, and ${\tilde{p}}_{2,r2}$. The detailed expressions for these corrections can be found elsewhere [26]. If we put $\frac{C_{T1}^{2}}{C_{T2}}=C_{T},\;$ where $C_{T}$ is called the “total correction”, combining Equations (11) and (12) yields the nonlinear parameter $\beta_{f}$ in fluids

(15) $$\beta_{f}=\frac{2\rho c^{2}}{kz}\frac{{\tilde{p}}_{2,r}}{{\tilde{p}}_{1,r}^{2}}C_{T}=\beta_{f}^{\prime }\;C_{T}$$

The nonlinear parameter $\beta_{s}$ in solids can be obtained by replacing $\beta_{f}$ with $\frac{1}{2}\beta_{s}$ in Equation (15). Then, using the relationship between pressure and displacement, $\beta_{s}$ can be determined in terms of the received average displacement by

(16) $$\beta_{s}=\beta =\frac{8}{k^{2}z}\frac{{\tilde{u}}_{2,r}}{{\tilde{u}}_{1,r}^{2}}C_{T}=\beta^{\prime }\;C_{T}$$

Since the amplitude of the actually measured wave deviates from the plane wave, $C_{T}$ appearing in Equation (16) is to correct the attenuation, diffraction, and boundary reflection effects in the received amplitudes of the fundamental and second harmonic waves. Hence, $\beta^{\prime }$ is called the “uncorrected” nonlinear parameter. 

## 4. Optimization of Phase Array Dimensions

The received second harmonic wave, ${\tilde{u}}_{2,r}$, in the pulse–echo testing is in general much smaller than the through-transmission method, consequently the uncorrected nonlinear parameter, $\beta^{\prime }$, becomes also very small. To recover the correct nonlinear parameter, *β*, a large value of the total correction, $C_{T}$, should be multiplied. For accurate and reliable determination of β, especially for thin specimens, it is necessary to maximize ${\tilde{u}}_{2,r}$ and reduce the dependence on $C_{T}$ by optimizing the design of APAT. These two parameters depend on many variables including sample thickness, frequency, and shape and size of the transmit and receive elements. Here, the thickness of the specimen is fixed, so it is not a design variable for optimization. It is also assumed that the frequency is fixed. Then, the optimization of APAT can be considered a process of determining the size, arrangement, and shape of the transmit and receive elements. The optimization of APATs can be approached in terms of two objective functions: the second harmonic reception and the total correction. The optimized transducer should provide the largest possible second harmonic reception and the total correction value as close to one as possible. 

In the simulation-based optimization here, the received amplitudes of the fundamental and second harmonics and the total correction were calculated through wave field analysis for various combinations of shape and size of the transmit and receive elements. Then, the optimized APAT is finally obtained by comparing the received second harmonic amplitude and the total correction value from various simulation cases.

As a final step, the waveform of the received signal was obtained through finite element analysis (FEA). The purpose of FEA is to check the distortion of waveforms received through focusing, and to validate the analytical model used for wave field calculation and optimization of annular phased arrays.

### 4.1. Focused Beam Field Analysis Results

APATs can be divided into two types: equal width (EW) and equal area (EA). The wave fields for these two types are analyzed and the focusing properties are compared. The source displacement used in the analysis is $u_{0}=10^{-9}\;m$, and the fundamental frequency is $f_{1}=5\;\mathrm{MHz}$. The attenuation effect is not considered. For the EW type, the element width is 1 mm and the kerf is 0.5 mm. For the EA type, the area of the innermost element is the same as the first element of the EW type, and the size of the remaining elements is determined from the area of the first element. The kerf is 0.5 mm. The number of elements in both types is four. The focal length (*F*) is set to 10 mm. The central receiver has a fixed diameter of 3.2 mm. The target thickness of the specimen is assumed to be 10 mm. The dimensions used in the wave field analysis are given in Table 2.

**Figure 6:**
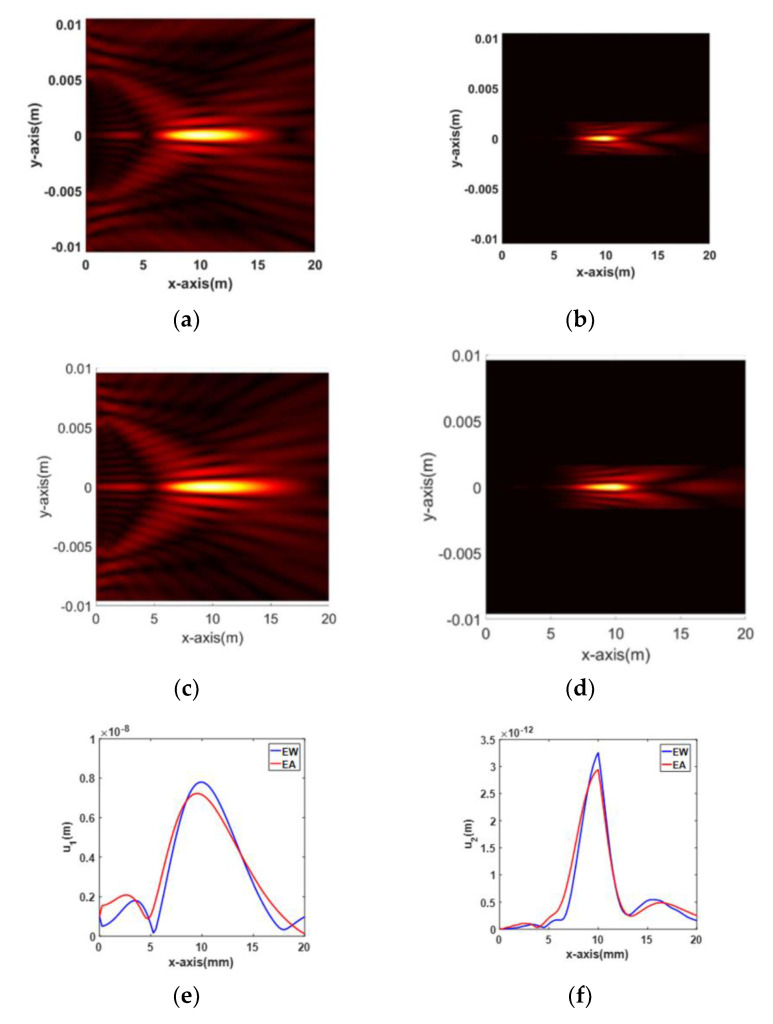
Simulation results and comparisons between the EW and EA types of APAT for fundamental and second harmonic waves: (**a**) fundamental, EW; (**b**) second harmonic, EW; (**c**) fundamental, EA; (**d**) second harmonic, EA; (**e**) on-axis, fundamental wave; and (**f**) on-axis, second harmonic.

Simulation results and comparisons between the EW and EA types of APAT for fundamental and second harmonic waves are shown in Figure 6. More specifically, the 2D beam profile and the on-axis variation of displacement are presented. When considering only the beam profile, there seems to be no difference in beam focusing between the two types. However, comparison of on-axis profiles shows a noticeable difference between the two types. The maximum amplitude of the EW type is slightly larger than that of the EA type at *F* = 10 mm in both wave types. The EA type is found to focus at a distance slightly shorter than *F* = 10 mm in the fundamental wave. Based on this, it can be said that the EW type has better focusing performance than the EA type. Therefore, optimization is performed using the EW type. 

The focusing behavior of the fundamental wave along the lateral and axial directions is shown in Figure 3. Similar behavior is also observed here, as shown in Figure 6a,c.

Compared to the fundamental wave, the second harmonic wave shows a narrower beamwidth at the focal length due to the twice as large frequency, as shown in Figure 6b,d. As a result, the second harmonic wave forms a sharp focus at the specified focal length. In fact, the amplitude of the received wave is determined by a receiver of finite size. Thus, in the case of a focused beam, it is important to determine the size of the receiver according to the focal spot size in the lateral direction of the beam in order to receive maximum amplitude. In Figure 6b,d, the focal spot sizes at −6 dB along the lateral direction are estimated to be less than 1 mm, which can be treated as a point focus. Therefore, for maximum reception of the second harmonic amplitude, a point reception device such as a laser interferometer may be the best choice, but, considering the actual situation, a broadband ultrasonic receiver made of the smallest possible size piezoelectric element may be more suitable.

### 4.2. Optimization for Second Harmonic Generation and Total Correction

In the EW type APAT, the focal length and element width affect the received amplitude of the second harmonic and the total correction. Here, the effect of these two parameters is examined. The focal position is set at three distances, namely 10, 15, and 20 mm, corresponding to the reflection boundary of the 10 mm thick specimen, the center of the specimen equal to 1.5 times the specimen thickness, and twice the specimen thickness equal to the receiver position. The width of the element is set to 1, 1.5, 2, and 2.5 mm. Twelve simulation cases are listed in Table 3. According to the simulation results in Section 4.1, among commercially available broadband transducers, a receiver with a minimum diameter of 3.2 mm is used.

**Figure 7:**
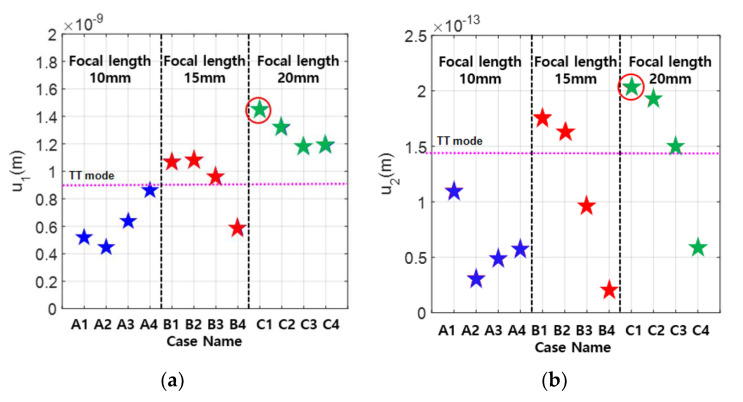
The received displacements calculated using the simulation parameters in Table 3: (**a**) fundamental wave; and (**b**) second harmonic wave.

Using various combinations of focal length and element width, the received fundamental and second harmonic amplitudes were calculated. The simulation results for 12 cases are shown in Figure 7. It also includes the results of the through-transmission (TT) mode calculations when a transmitter and a receiver both 12.7 mm in diameter are used. The propagation distance for the single element TT mode is 10 mm, which is the thickness of the specimen. The received amplitude is largest in Case C-1 for both the fundamental and the second harmonic waves. This result shows that the optimal design of APAT with the beam focusing can produce a received second harmonic amplitude that is about 50% larger than the single element TT case.

**Figure 8:**
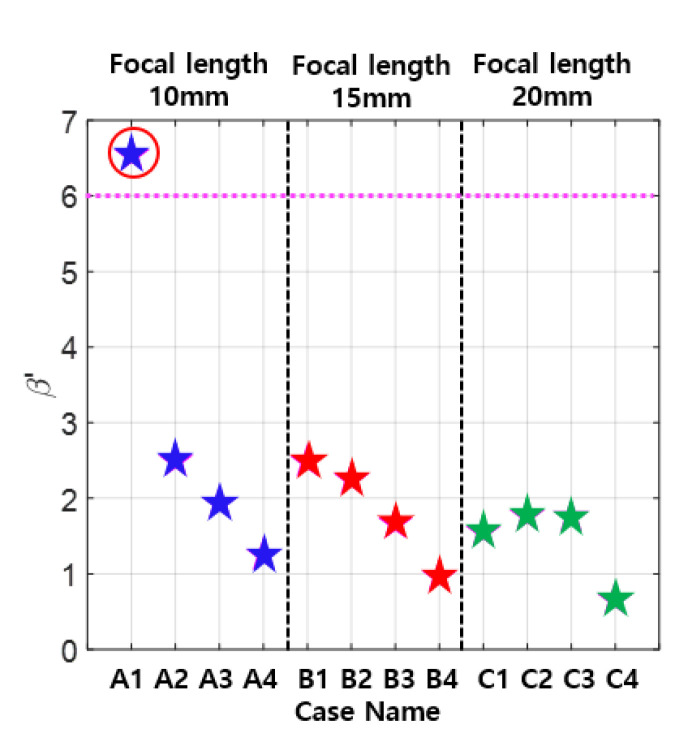
The calculated *β*’ using the data in Figure 7.

The uncorrected $\beta^{\prime }$ was calculated using the received amplitude data in Figure 7, and the results are shown in Figure 8. Since the nonlinear parameter is given by $\beta =\left[\beta ’\right]\left[C_{T}\right]$, the total correction $C_{T}$ should be as close to one as possible in the optimization process. This means that the uncorrected $\beta^{\prime }$ should be as close to β as possible. It can be seen that $\beta^{\prime }=1.56$ in Case C-1, where the received second harmonic amplitude is the largest, and $\beta^{\prime }=2.53$ in Case B-1. Therefore, the optimal case for $\beta^{\prime }$ or $C_{T}$ is Case A-1 giving $\beta^{\prime }=6.55,$ which is about 19% larger than β=5.5. These results show that the APAT specifications optimized for one objective function may not satisfy the other objective function.

Next, the influence of kerf was analyzed by changing the kerf size to 0.1, 0.3, and 0.5 mm in the EW type APAT. When designing an APAT, the interelement spacing (kerf) should be less than half the wavelength to suppress the occurrence of grating lobes. All three kerf sizes selected here meet this condition. Three different focal lengths were used as before. Various combinations of focal length and kerf size are shown in Table 4. 

**Figure 9:**
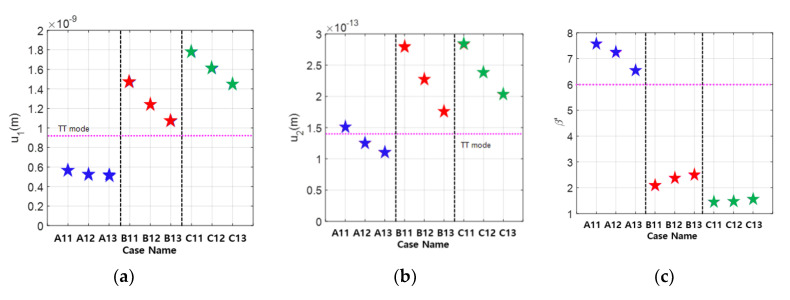
Received displacements and uncorrected nonlinear parameter calculated using the simulation parameters in Table 4: (**a**) fundamental wave; (**b**) second harmonic wave; and (**c**) uncorrected nonlinear parameter.

The simulation results for various combinations of data in Table 4 are shown in Figure 9, showing the received fundamental and second harmonic amplitudes and the uncorrected $\beta^{\prime }$. From the viewpoint of the maximum second harmonic reception, the best case is B11 or C11, and, from the viewpoint of $\beta^{\prime }$ or $C_{T}$, Case A13 may be better. Considering both of these goals, all three cases in Group A are good. These results show that the APAT specifications optimized for one objective may not satisfy the other objective. Therefore, in the optimization of the APAT specification, the objective function—second harmonic reception, total correction, or both—needs to be clearly defined.

### 4.3. Summary of Optimization Results

In relation to the measurement of nonlinear parameters of materials in the pulse–echo mode, the optimal design of the annular phased array transmitter consisting of four equal width (EW) elements was considered. With the specimen thickness, frequency, and receiver size fixed, the optimization of the APAT was performed from two viewpoints: received second harmonic amplitude and total correction. The received amplitudes of the fundamental and second harmonics and the total correction were calculated through wave field analysis for various combinations of the element width, kerf, and focal length of the transmitter. Then, the optimized specifications of the APAT were obtained by comparing the received second harmonic amplitude and the total correction from various simulation cases. In the optimal design process of APAT, the results of the through-transmission (TT) method by a single transmitter and a single receiver were used as a reference.

The optimization results are summarized in Table 5, where the three optimized APAT designs—A13, B13, and C13 in Table 4—are given together with the TT results. The kerf sizes of these types are all 0.5 mm, and the focal length is 10, 15, and 20 mm, respectively. From the viewpoint of the maximum second harmonic reception only, the best case is B13 or C13, and, from the viewpoint of uncorrected nonlinear parameter $\beta^{\prime }$ or the total correction $C_{T}$ only, A13 is better. Considering both of these conditions, A13 is just fine. These results show that the APAT specifications optimized for one objective may not satisfy the other objective. Therefore, in the optimization of the APAT specification, the objective function—second harmonic reception, total correction, or both—should be clearly specified.

We already developed the measurement procedure to determine material nonlinearity in the pulse–echo method using a single element transducer and a dual element transducer [29,36,37]. The current work is the extension of our previous work on the dual element transducer approach. The single annular transmit element was simply replaced by the four annular transmit elements to create a focused beam at a specific location in the specimen. If the annular phased array transducer with four element transmitter and a single element receiver can be made and used, similar measurement procedures can be applied, including receiver calibration. 

### 4.4. FE Simulation Results

The analytical acoustic model introduced in Section 3 is a method of calculating the wave field in the frequency domain and provides the received displacement value at a specific frequency. To obtain the received waveform in the time domain, displacement must be calculated at hundreds of frequency values and then inversely Fourier-transformed. Therefore, the analytic method is not suitable for time domain waveform calculation. In ultrasonic modeling, one of the most efficient ways to directly calculate the waveform is the finite element method. 

In the case of performing a nonlinear experiment using the optimized APAT of Section 4.1 and Section 4.2, a tone burst waveform of tens of cycles is used as an input signal and is received by the receive transducer after being focused on a specific position in the specimen. Therefore, it may be necessary to ensure that the time-delayed signal emitted by each element of the APAT is arrived in-phase at the focal position and then received in the same waveform as the initially incident signal without distortion. 

In this section, the waveform of the received signal was obtained through FEA. The purpose of FEA is to check the distortion of the received waveform after being focused on a position in the specimen, and to validate the analytical model used for wave field calculation and optimization of annular phased arrays. COMSOL Multiphysics FE program was used to simulate the nonlinear wave fields calculation. The specimen used is an aluminum with quadratic material nonlinearity [39]. The quadratic nonlinear material is defined by the third-order elastic constants *l*, *m*, and *n*, which is also called “Murnaghan material” in the built-in option of COMSOL program. Simulation was carried out using the second-order axisymmetric model. The source displacement used in the FE simulation was $u_{0}=10^{-7}\;m$, which is two orders of magnitude larger than that used in the analytical simulation. This is for easy visualization of the relatively small second harmonic component in the received signal of the FE simulation.

**Figure 10:**
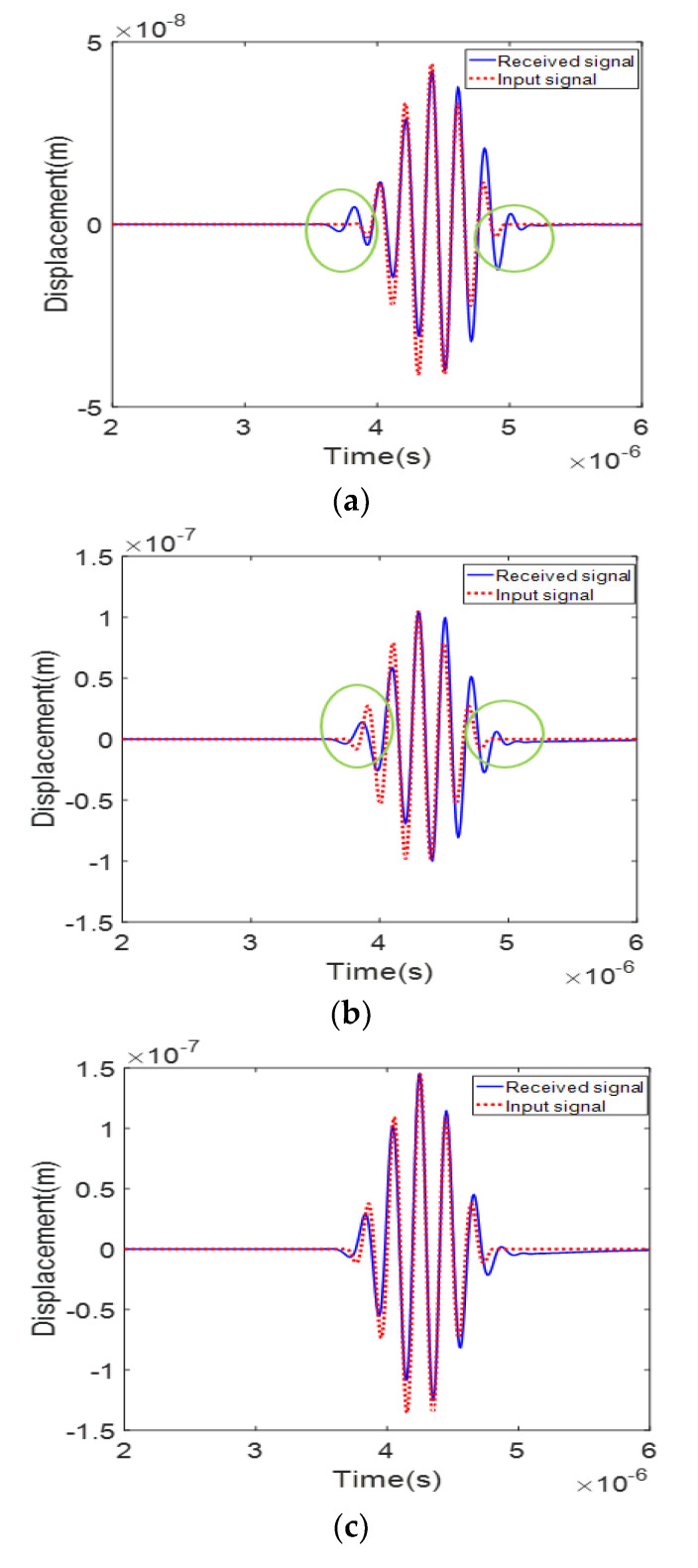
The received signal waveforms for three different APAT cases in Table 4: (**a**) Case A13; (**b**) Case B13; and (**c**) Case C13.

The three types of EW APAT in Table 4—A13, B13, and C13—were used in the FE simulation. The kerf sizes of these types are all 0.5 mm, and the focal length is 10, 15, and 20 mm, respectively. The FE simulation results are presented in Figure 10, showing the received signal waveforms for the three cases. The results show that the peak amplitude of the received waveform, measured in the order of C13 > B13 > A13, is in good qualitative agreement with the analytical simulation results shown in Figure 9a. In the case of C13, where the focal position and the receiver position are the same (Figure 10c), the signal radiated from each element appears to arrive in-phase at the specified focal position. In addition, the overall shape of the waveform seems to match the input signal well without any significant difference. In Cases A13 and B13, where the focal position and the receiver position are different, there is a slight difference in the leading and trailing edges of the received waveform compared to the input waveform. This difference may occur because the focal position and the reception position are not the same, and it occurs slightly larger in Case A13, where the difference between these two positions is larger. Since the difference in waveform is very small, it is believed that it will have little effect on the measurement of nonlinear parameters. If the reception time delay is applied to the received waveform, the waveform difference can be reduced.

However, it was determined that there is little effect on the measurement of nonlinear parameters because the distortion of the waveform was not large in the simulation results.

**Figure 11:**
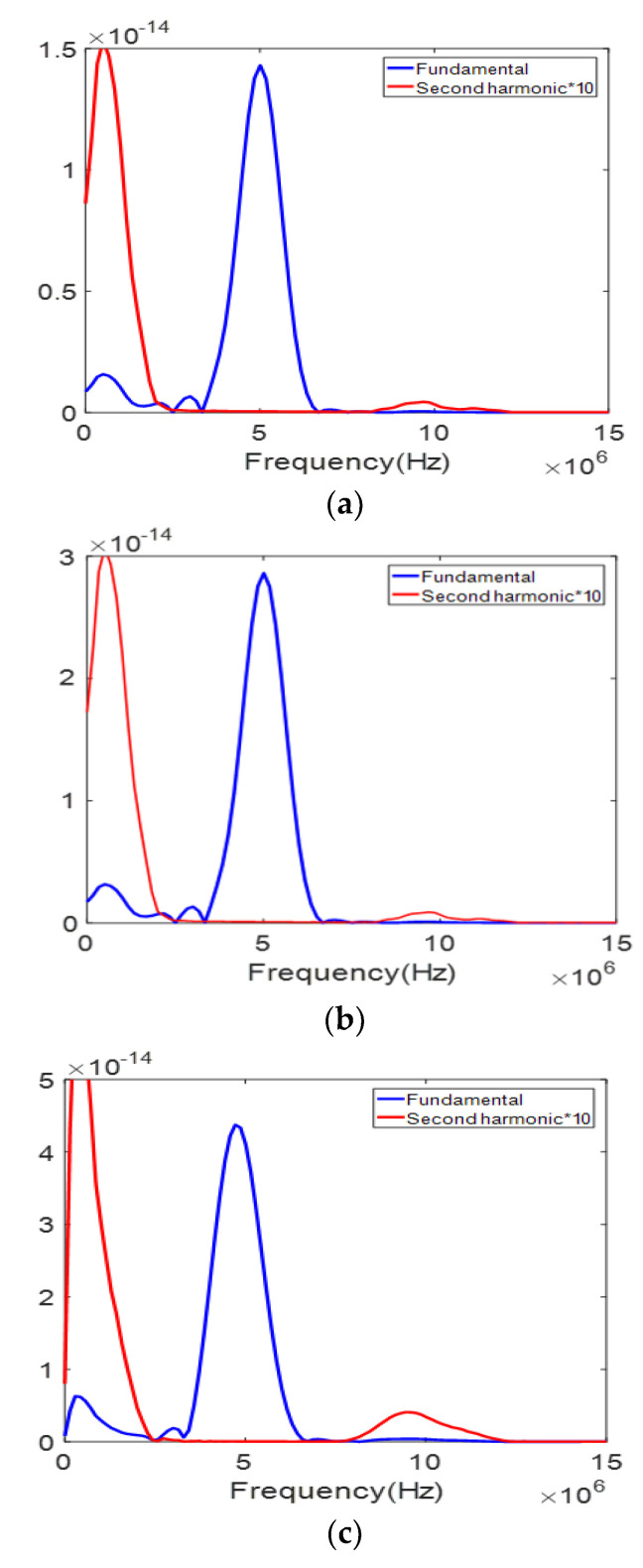
The received signal spectrum for three different APAT cases in Figure 10: (**a**) Case A13; (**b**) Case B13; and (**c**) Case C13.

The frequency spectrum of the received signal in Figure 10 is shown in Figure 11. To easily visualize the second harmonic component, the spectral values were multiplied by 10. The second harmonic component can be clearly seen in all three cases. The peak magnitude of each spectrum is in the order of C13 > B13 > A13, which also agrees well with the analytical calculation results. It should be noted that a spectral component of large size is present in the very low frequency region. This is known as a zero-frequency component or a quasi-static component which is produced by nonlinear acoustic wave propagation in an elastic solid of quadratic nonlinearity. Although its existence has been proven through theory and FE simulation [39,40], it is not easy to experimentally observe this component because a wideband receiver that covers down to zero frequency cannot be easily found.

## 5. Conclusions

In this paper, we present the analytical model, optimization method, and optimized design results of annular phased array transmitter for efficient second harmonic generation and nonlinear parameter determination in the pulse–echo nonlinear ultrasonic testing. The annular phased array transducer consisting of four-element transmitter and a single-element receiver was optimized in terms of second harmonic reception and total correction. The performance of various combinations of transmitter design variables and focal lengths were tested through wave field analysis, and the optimized specifications of the transmitter were determined and presented. In the future, the fabrication and experimental verification of optimized annular phased array transducers through acoustic performance testing and nonlinear parameter measurement is required. In addition, when using the focused beam of the annular phased array transducer, applying a reception time delay will further enhance the received second harmonic amplitude in the pulse echo mode. These additional studies are expected to develop the pulse–echo nonlinear ultrasonic tests as more practical nondestructive evaluation and diagnosis techniques.

## Figures and Tables

**Table 1:** Specifications of linear and annular phased arrays used in the simulation.

Array Pattern	Linear	Annular
Number of elements	8	4
Gap between elements	0.5 mm	0.5 mm
Element width	1 mm	1 mm
Element length	10 mm	-
Total array width	21 mm	21 mm

**Table 2:**
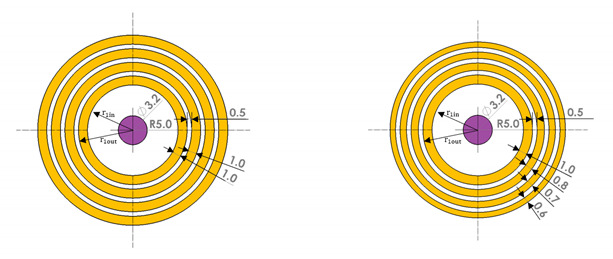
Dimensions of two types of annular phased arrays (unit: mm).

EW				EA			
Radius		Radius		Radius		Radius	
$r_{1in}$	5	$r_{1out}$	6	$r_{1in}$	5	$r_{1out}$	6
$r_{2in}$	6.5	$r_{2out}$	7.5	$r_{2in}$	6.5	$r_{2out}$	7.3
$r_{3in}$	8	$r_{3out}$	9	$r_{3in}$	7.8	$r_{3out}$	8.5
$r_{4in}$	9.5	$r_{4out}$	10.5	$r_{4in}$	9	$r_{4out}$	9.6

**Table 3:** Simulation cases for various combinations of focal length and element width.

Group	Case Number	Focal Length (mm)	Element Width
A	1	10	1
	2		1.5
	3		2
	4		2.5
B	5	15	1
	6		1.5
	7		2
	8		2.5
C	9	20	1
	10		1.5
	11		2
	12		2.5

**Table 4:** Simulation cases for various combinations of focal length and kerf size.

Case Numbers	Focal Length (mm)	Kerf (mm)
A11	10	0.1
A12		0.3
A13		0.5
B11	15	0.1
B12		0.3
B13		0.5
C11	20	0.1
C12		0.3
C13		0.5

**Table 5:** Summary of optimized APAT specifications and simulation results.

Specification	Single (TT)	PA (PE)	PA (PE)	PA (PE)
Transmitter (mm)	Diameter = 12.7	Element width = 1 kerf = 0.5		
Focal length (mm)	-	10	15	20
Receiver dia. (mm)	12.7	3.2	3.2	3.2
$u_{1}$(m)	$8.82\times 10^{-10}$	$5.20\times 10^{-10}$	$1.07\times 10^{-10}$	$1.45\times 10^{-9}$
$u_{2}$(m)	$1.46\times 10^{-13}$	$1.10\times 10^{-13}$	$1.76\times 10^{-13}$	$2.03\times 10^{-13}$
$\beta^{\prime }$	6.04	6.54	2.48	1.56
$C_{T}$	0.91	0.84	2.22	3.53

Note: PA = Phased array; TT = Through–transmission; PE = Pulse-echo.
